# Amplification of Ultra-Trace DNA from Early Sheep Embryos Based on qPCR: Establishing a Gender Identification System

**DOI:** 10.3390/biology14091144

**Published:** 2025-08-29

**Authors:** Peng Niu, Weikun Tao, Fei Huang, Xiaopeng Li, Xueyan Wang, Jie Wang, Qinghua Gao, Di Fang

**Affiliations:** 1 College of Life Science and Technology, Tarim University, Alar 843300, China; 10757203053@stumail.taru.edu.cn (P.N.); 107572022315@stumail.taru.edu.cn (F.H.); miexiaochi@163.com (X.L.); wangxueyan1016@163.com (X.W.); 120240063@taru.edu.cn (J.W.); 119920009@taru.edu.cn (Q.G.); 2 College of Animal Science and Technology, Shihezi University, Shihezi 832003, China; taoweikun@hotmail.com; 3 Key Laboratory of Tarim Animal Husbandry Science and Technology, Xinjiang Production & Construction Corps, Alar 843300, China

**Keywords:** *SRY*, *GAPDH*, probe, qPCR, embryo, sex identification

## Abstract

The ability to identify and control the sex of embryos holds significant commercial implications for livestock production. Due to the high costs and instability of detection methods, it is necessary to establish a time-saving, effective, simple, and reliable method to identify the sex of mammalian embryos. This study designs primer probes based on the *SRY* gene sequence in the sex-determining region of the Y chromosome of sheep and combines qPCR to amplify both the *SRY* gene and the internal reference gene *GAPDH* as a control. By adjusting and optimizing the detection system with ultra-trace of genomic DNA (gDNA) from sheep blood and embryos, we sexed sheep embryos cultured in vitro. The results show that male embryos exhibit specific amplification bands for both the *GAPDH* and *SRY* genes, while female embryos only have amplification bands for the *GAPDH* gene. The sensitivity of the sheep embryo sex identification system established in this study can be as low as 5.64 pg of DNA, with an accuracy rate of 100% for embryo sex identification.

## Simple Summary

Identifying the sex of early mammalian embryos is key for gender control. This study used ultra-trace amounts of sheep embryonic DNA templates and specific probes to amplify the *SRY* gene, establishing a time-saving, effective, simple, and reliable embryo sex identification method. The results showed that the accuracy of embryo sex identification was 100%. Combined with embryo transfer, this technology can accelerate breed selection and promote the development of embryo commercialization.

## 1. Introduction

In livestock production, many production traits are controlled by sex, and pre-selecting the sex of offspring guarantees improvement in the production efficiency and sustainability of the livestock industry. As important economic industries, dairy and poultry egg processing benefit from a higher female-to-male sex ratio when increasing output [1]. At the same time, selectively producing females is advantageous for optimizing the reproductive potential of parent stocks. Some human genetic diseases are transmitted through sex-related patterns [2,3]; therefore, developing technology to pre-select the sex of offspring is of great practical value for precise breeding management, promoting genetic improvement, and reducing the spread of sex-related diseases.

In recent years, technological innovation based on embryonic sex determination has accelerated, including single-cell sequencing, pre-implantation genetic diagnosis, non-invasive DNA testing, ultra-low temperature preservation, and embryo transfer [4,5,6]. Studies have shown that male and female mammalian embryos differ not only in chromosomal complement but also in transcriptional activity and epigenetic state. Sexual dimorphism in embryonic transcription affects carbohydrate and amino acid metabolism, with female embryos showing slower mitochondrial metabolism [7], providing new ideas for molecular markers combined with omics analysis for embryo sex identification. Herr et al. first used PCR methods for sex identification in bovine embryos [8]. Polisseni et al. optimized the time, accuracy, and stability of embryo sex identification, and this method has since been widely adopted [9]. However, current research predominantly focuses on the amplification efficiency of high-concentration genomic DNA (gDNA), while studies examining the amplification and detection of low-concentration gDNA, particularly at ultra-trace levels, remain relatively scarce.

The *SRY* gene is the main effective gene on the Y chromosome’s sex-determining region. It is composed of a single exon, encoding a protein structure domain with a high-mobility group (HMG) that binds to DNA and shows high sequence conservation [10]. The characteristics of the *SRY*-HMG make it suitable for developing a DNA sex determination test [11]. *SRY* is only expressed in males, so the absence of *SRY* gene amplification may result in false-positive outcomes. Glyceraldehyde 3-phosphate dehydrogenase (GAPDH) is an enzyme encoded by an autosomal gene, converting glucose into pyruvate in the glycolytic pathway [12]. Researchers have discovered that the *GAPDH* gene is highly conserved across mammals and is frequently employed as a classical reference gene in species such as cattle, sheep, horses, and chickens [13,14,15,16]. Therefore, amplifying the *SRY* and *GAPDH* genes, combined with fluorescence in situ hybridization (FISH), has been utilized for sex identification [17,18].

This study selects the *SRY* gene sequence and specific probes from rams to design primers and constructs a composite detection system by combining two sets of these primers. We aim to establish a method for identifying the sex of sheep embryos using ultra-trace embryonic DNA, thereby addressing the identified research gap. This avoids the potential false negatives that may occur if the *SRY* gene is not amplified in female embryos, improving the sensitivity and identification efficiency of the results.

We propose the following hypothesis: by optimizing qPCR amplification conditions, we can establish an efficient ultra-trace gDNA amplification system for sheep embryos, enabling accurate gene detection at minimal gDNA concentrations. Initially, we established an optimal identification system using sheep blood gDNA and evaluated its specificity at the lowest dilution factor through qPCR amplification. Ultimately, this method was employed to determine the sex of sheep embryos.

## 2. Materials and Methods

### 2.1. Ethics Statement

All animal experiments were conducted following the “Regulations and Guidelines for the Management of Experimental Animals” established by the Ministry of Science and Technology (Beijing, China, 2020 revision). This study was approved by the Institutional Animal Care and Use Committee of Tarim University, Xinjiang, China (protocol code DWBH20240814; approval date: 14 August 2024).

### 2.2. Experiment Location and Time

Experiments were conducted in Alar, China, Xinjiang (40°54′ N, 81°30′ E). Healthy sheep with an average age of 3 years were selected for the experiment. Adequate forage, management, and light intensity were ensured. The sheep were free to drink and supplemented with trace elements, and no other antioxidants were added to their forage.

### 2.3. Extraction of Blood gDNA

Venous blood samples from sheep (3 males and 3 females) were collected and preserved in anticoagulant tubes. Genomic DNA was extracted from the blood using a Transgen Biotech kit (Beijing, China; technical replicates *n* = 3). A nucleic acid detector (Nano-400A, Beijing, China) was used to measure the blood DNA concentration (males: 112.8 ± 3.2 ng/μL, females: 113.8 ± 2.9 ng/μL). The blood DNA was diluted in a gradient of 10, 100, 1000, 10,000, and 20,000 times with ddH_2_O, and the concentrations were measured and stored at −20 °C (males: 11.28 ± 0.21 ng/μL; females: 11.38 ± 0.18 ng/μL), (males: 1.128 ± 0.03 ng/μL; females: 1.138 ± 0.04 ng/μL), (males: 0.1128 ± 0.56 × 10^−2^ ng/μL; females: 0.1138 ± 0.83 × 10^−2^ ng/μL), (males: 0.01128 ± 0.74 × 10^−3^ ng/μL; females: 0.01138 ± 0.98 × 10^−3^ ng/μL), (males: 0.00564 ± 0.41 × 10^−3^ ng/μL; females: 0.005569 ± 0.46 × 10^−3^ ng/μL).

### 2.4. Extraction of Embryo gDNA

#### 2.4.1. Embryo Production System

Sheep ovaries were collected from the Aksu slaughterhouse (Permit No. XJ-2023-092) and transported in sterile Phosphate-Buffered Saline (PBS; Saiweier Co., Ltd., Wuhan, China) supplemented with 200 IU/mL penicillin–streptomycin under controlled temperatures (38 ± 0.5 °C) within 2.5 ± 0.3 h.

#### 2.4.2. Oocyte Maturation

Cumulus–oocyte complexes (COCs) were aspirated from 2 to 6 mm follicles using 18 G needles (Kangjian Medical, Taizhou, China, KJ-1820). The grading of COCs is based on their morphological characteristics. Grade A is defined by the presence of at least three layers of tightly arranged cumulus cells, showing no signs of degeneration or expansion. Grade B is characterized by fewer than three layers of cumulus cells, with a uniform oocyte cytoplasm. Grade C includes oocytes that are not surrounded by cumulus cells and may exhibit uneven granulation in the cytoplasm. Finally, Grade D is marked by significant signs of degeneration in the oocytes, including complete degeneration or the absence of cumulus cells. In this experiment, A/B grade COCs were selected for maturation, with 5% estrous sheep serum, 1 mM sodium pyruvate, 1.8 µg/µL heparin, 14 µM isoproterenol, and 2 mg/mL BSA (Solarbio Co., Ltd., Beijing, China) added to an SOF medium (Huankai Microbial, Guangzhou, China). This medium is manufactured by Sigma-Aldrich (St. Louis, MO, USA), a German company, and is identified by the product number M8776. Incubation parameters: 38.5 °C, 5% CO_2_, 100% humidity for 23 ± 0.5 h.

#### 2.4.3. Sperm Preparation

Fresh sheep semen was collected and transported to the laboratory at a constant temperature of 38 °C. It was then diluted with Bicarbonate-Optimized buffer (BO buffer; Solarbio Co., Ltd., Beijing, China) at a 1:3 volume ratio and centrifuged at 800× *g* for 5 min. The semen sample was transferred to a 15 mL conical tube. The tube wall was carefully covered with 2 mL of capacitation medium (SP-TALP buffer; Huankai Microbial, Guangzhou, China) and incubated at 38.5 °C in a 5% CO_2_ environment for 45 min. Following incubation, we aspirated the upper 80% of the liquid, which contained the highly motile sperm [19].

#### 2.4.4. Embryo Culture

The mature oocytes were washed three times with fertilization medium and subsequently transferred into 50 μL of pre-equilibrated fertilization medium, ensuring that a maximum of 15 COCs were added per drop. The upstream semen was added to the fertilization droplets (the sperm density was approximately 1 × 10^6^/mL). The sperm and oocytes were incubated in a CO_2_ incubator for 18 h (2% essential amino acids, 1.2 mM sodium pyruvate, NaHCO_3_ (Saiweier Co., Ltd., Wuhan, China), and 4% BSA were added to the SOF medium.

#### 2.4.5. Embryo Biopsy Protocol

Morula-stage embryos were biopsied using an Eppendorf TransferMan^®^ 4 micromanipulator mounted on an Olympus IX73 inverted microscope (Olympus Corporation, Tokyo, Japan). We utilized a holding pipette (Origio MXL3-100—outer diameter, 100 µm; inner diameter, 25 µm) to securely position the embryo, followed by a biopsy with a biopsy needle (Humagen MIC-20; inner diameter, 20 µm; Nikon Co., Ltd., Shenzhen, China).

#### 2.4.6. gDNA Extraction

Biopsied cells (5–8 inner cells per embryo) were lysed in 56 °C lysis buffer containing the following: 0.5% SDS (Sigma, L3771; St. Louis, MO, USA), 200 μg/mL Proteinase K (ThermoFisher, EO0491; Waltham, MA, USA), and 10 mM Tris-HCl (pH 8.0). Thermal cycling: 95 °C for 10 min, ice quenching (2 min), centrifugation (2000× *g*, 10 min). The gDNA was diluted in a gradient of 1.5, 1.2, and 1 μL with ddH_2_O, and the concentrations were measured and stored at −20 °C: (males: 22.89 ± 1.32 pg/μL; females: 22.96 ± 1.28 pg/μL), (males: 11.42 ± 1.14 pg/μL; females: 11.48 ± 1.12 pg/μL), (males: 5.78 ± 0.65 pg/μL; females: 5.86 ± 0.71 pg/μL).

#### 2.4.7. Cryopreservation

Another part of the morula-stage embryos was frozen using an embryo freezer (CL-5500, Guangzhou, China): the program started at 20 °C for 28 min (cooling rate 0.61 °C/min), −7 °C for 10 min (2.70 °C/min), −30 °C for 74 min (0.31 °C/min), −33 °C for 30 min (0.10 °C/min). Then, the sample was transferred to liquid nitrogen for storage.

### 2.5. Primer and Probe Design and Synthesis

The *SRY* and *GAPDH* genes sequences of the sheep Y chromosome sex-determining region (*SRY*, GenBank accession No: Z30265; *GAPDH*, GenBank accession No: NM-001190390) were designed using BLAST (Blastn, 2.14.0) and PREMIER (Premiere Pro 2020, 14.0) to create primers and probes (Table 1). Primers and probes were synthesized by New Bei Biological Technology Co., Ltd., Shanghai, China.

### 2.6. Blood qPCR Reaction System

This study utilized the Bio-Rad system (CFX96, Hercules, CA, USA) for qPCR experiments. *SRY* gene: 5 μL of 2 × S6Probe qPCR Mix, 0.2 μL each of forward and reverse primers (concentration 10 μmol/L), 0.1 μL of probe (5 μmol/L), 1 μL of template DNA (dilution concentrations at gradient 2.3), and ddH_2_O added to a final volume of 10 μL. *GAPDH* gene: 5 μL of 2×S6Universal SYBR qPCR Mix, 0.25 μL each of forward and reverse primers, 1 μL of template DNA, and ddH_2_O added to a final volume of 10 μL. qPCR reaction conditions: initial denaturation at 95 °C for 3 min, followed by 45 cycles of denaturation at 95 °C for 20 s, annealing at 60 °C for 20 s, and a final extension at 72 °C for 5 min, with the reaction products held at 4 °C.

### 2.7. Embryo Ultra-Trace qPCR Reaction System

*SRY* gene: gradient concentrations of gDNA at 1.5, 1.2, 1 μL, and 0.2 μL each of forward and reverse primers (dilution concentrations at gradient 2.4), 5 μL of 2 × S6Probe qPCR Mix, and ddH_2_O added to a final volume of 10 μL. *GAPDH* gene: 5 μL of 2 × S6Universal SYBR qPCR Mix, 0.25 μL each of forward and reverse primers (10 μmol/L), 1 μL of template DNA, and ddH_2_O added to a final volume of 10 μL. qPCR reaction conditions: initial denaturation at 95 °C for 3 min, followed by 45 cycles of denaturation at 95 °C for 20 s, annealing and extension at 60 °C for 20 s, and a final extension at 72 °C for 5 min, with the reaction products held at 4 °C.

### 2.8. Agarose Gel Electrophoresis

The qPCR amplification products (4 μL) were thoroughly mixed with the electrophoresis buffer (1 μL) and then added to the wells of a 2% agarose gel in sequence. Electrophoresis conditions: 150 V voltage, 130 mA current, 20 min. The results were observed using a gel imaging system. The amplification results for males showed two bands, while females had only one band.

### 2.9. Data Analysis

qPCR data was analyzed utilizing Bio-Rad’s CFX Manager (Version 3.1), while the real-time quantitative qPCR data were evaluated using the 2^−ΔΔCt^ method. Furthermore, statistical analysis was performed using GraphPad Prism version 9.5.1.

## 3. Results

### 3.1. Establishing the Blood gDNA Ultra-Trace qPCR Amplification System

**Figure 1:**
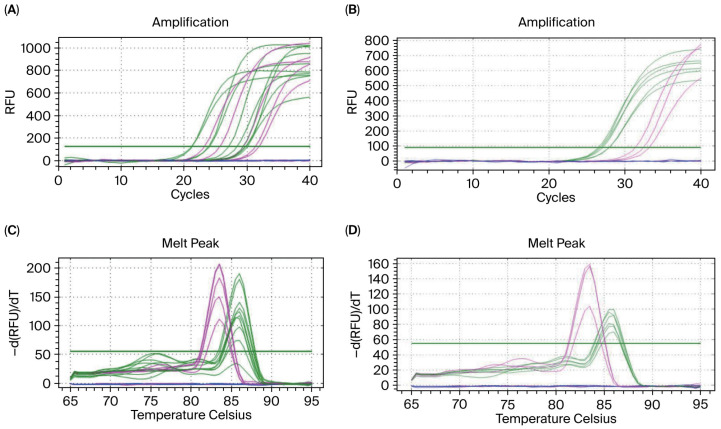
Establishment of sheep blood ultra-trace gDNA amplification system. Note: (**A**) Amplification curves at dilution factors of 10, 100, 1000, 10,000, and 20,000 times; (**B**) melting curves corresponding to the same dilution factors; (**C**) validation results for the qPCR amplification curve at the maximum dilution factor of 20,000 times; (**D**) validation results for the qPCR melting curve at the same maximum dilution factor. The green curve represents the *GAPDH* gene, the pink curve represents the *SRY* gene, and the blue curve indicates the blank control group. In addition, the Y-axis RFU represents the Relative Fluorescence Unit, while -d(RFU)/dT indicates the rate of change of the fluorescence signal concerning temperature.

Sheep blood gDNA was diluted in a gradient of 10, 100, 1000, 10,000, and 20,000 times to obtain amplification curves, as shown in Figure 1A. All curves were stable, and the smaller the DNA concentration, the higher the ct (threshold cycle) value. The male sheep blood gDNA sample demonstrated amplification curves of the *GAPDH* and *SRY* genes, while the female sheep blood gDNA sample only demonstrated the amplification curve of the internal reference gene, *GAPDH*. The results were consistent with the experimental design.

The melting curve is demonstrated by amplifying sheep blood group DNA samples with gradient concentrations is shown in Figure 1B. The Tm values of the *GAPDH* and *SRY* genes were 86 °C and 83.5 °C, respectively. The melting curve was a single peak, and there was no primer dimer or non-specific amplification.

### 3.2. Verifying the Amplification Results for Blood gDNA Diluted 20,000 Times

The qPCR amplification curves of sheep blood gDNA diluted 20,000 times (♂5.64 pg/μL, ♀5.69 pg/μL) are shown in Figure 1C. Each sample was amplified in triplicate, and the detection accuracy of the *SRY* gene in the samples was 100%. The melting curve is shown in Figure 1D, and the results are completely consistent with the experimental design.

### 3.3. Serial Dilution of Blood gDNA for Electrophoresis Detection

**Figure 2:**
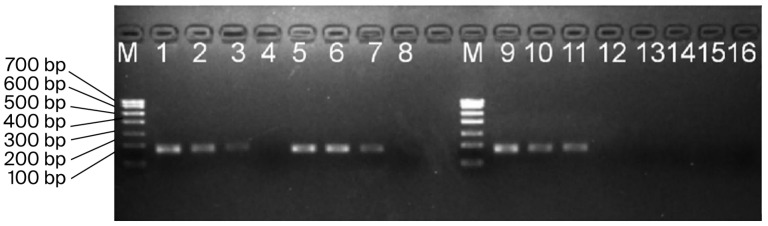
Gradient-diluted gDNA detected through agarose gel electrophoresis. Note: The dilution multiples are 10/100/1000, and lane M is the DNA marker. The gDNA electrophoresis results for male sheep are as follows: lanes 1/2/3 are the *GAPDH* gene; lanes 5/6/7 are the *SRY* gene; the gDNA electrophoresis results for female sheep are as follows: lanes 9/10/11 are the *GAPDH* gene; lanes 13/14/15 are the *SRY* gene. Lanes 4/8/12/16 are the blank controls.

The gradient concentration template amplification products of the blood gDNA were subjected to electrophoresis detection, and the results are shown in Figure 2. Males exhibited 162 bp *SRY* and 167 bp *GAPDH* gene amplification bands, while females only had a 167 bp *GAPDH* gene amplification band. The brightness of the amplification bands dimmed with decreasing concentrations of the amplified samples. The electrophoresis results confirmed the accuracy of the qPCR amplification results.

### 3.4. Gradient Concentration Amplification Results for Embryo gDNA

**Figure 3:**
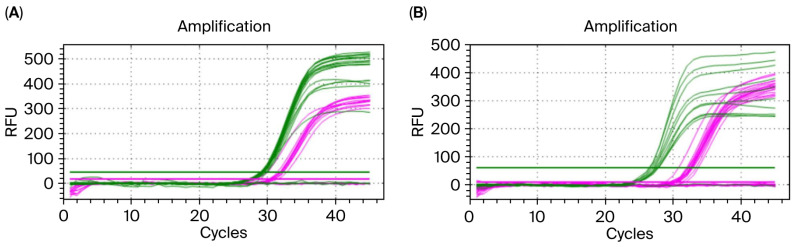
Sex identification based on sheep morula gDNA via qPCR amplification. Note: (**A**) qPCR amplification system for embryo gDNA gradient dilution (1.5, 1.2, 1 μL) is established; (**B**) amplification results for sex identification using ultra-trace of sheep embryo gDNA; the green curve represents the *GAPDH* gene, and the pink curve represents the *SRY* gene.

In this experiment, 1.5 μL, 1.2 μL, and 1 μL of gDNA solution were added to the qPCR reaction system. The results of the qPCR amplification are presented in Figure 3A. All six embryos amplified the *GAPDH* gene, and four embryos amplified the *SRY* gene, indicating they were male. Meanwhile, the optimal amplification curve was obtained when the volume of the gDNA solution was 1 μL.

### 3.5. Embryo Sex Identification

The experimental results concerning embryo sex identification show that ten representative embryos were randomly selected for analysis. The qPCR amplification results are illustrated in Figure 3B. All ten embryos amplified the *GAPDH* gene; six male embryos showed the *SRY* gene amplification curve, while four female embryos did not show the *SRY* gene amplification curve, with 100% identification accuracy.

## 4. Discussion

When combined with superovulation and embryo transfer techniques, sex control technology plays a crucial role in livestock production. As a primary method of sex control, embryo sex identification enables the production of offspring of a desired sex through embryo transfer, significantly contributing to improving production technologies and providing economic benefits [20]. This study established a qPCR amplification reaction system for ultra-trace gDNA in sheep’s blood and further adjusted the annealing temperature and cycle number to optimize the reaction system. The results show that the identification results are accurate and reliable under the following conditions: 20 μL of the amplification system, an annealing temperature of 60 °C, and 45 cycles. Nine sheep embryos were selected for gender identification using these methods, with a sensitivity of up to 5.64 pg and 100% accuracy. This method proved to be more sensitive and accurate than the findings of previous research [21,22,23].

Research shows that qPCR technology can quickly identify early embryos [24]. Mammalian Y chromosomes contain *SRY*-specific genes, which have high homology and high conservatism [25]. The sex identification protocol and primers used in this experiment are cheaper and more efficient than other methods described in the introduction, mainly for determining the sex of embryo biopsies before embryo transfer. The specific expression of *SRY* in male embryos is sufficient to determine their gender and has been used in different studies [26,27,28,29]. However, given the absence of the *SRY* gene in female embryos, it is not yet certain whether this indicates a positive result or PCR failure. Therefore, we designed *SRY*-specific probes, showing that the combined amplification of the housekeeping gene *GAPDH* can avoid the misinterpretation of false-negative results.

Ensuring reaction specificity with the optimal annealing temperature for specific primers is a critical step in improving the efficiency of qPCR amplification [30]. We designed temperature gradients and explored their effects multiple times, ultimately selecting an optimal annealing temperature of 60 °C. In addition, combining embryo gender identification with transplantation technology can provide greater benefits. This study conducted biopsies on embryos at the morula stage, employing micromanipulation instruments to extract 5–8 inner cells from each embryo. This procedure is widely used in preimplantation genetic diagnosis (PGD) and has been shown to be effective in obtaining genetic material for analysis [9,31].

Through blood tests, researchers such as María Sánchez and Rogers have found that the *SRY* gene product is only produced in ewes carrying male lambs, indicating that the detection time can only occur after the fetus has established contact with the mother [32,33]. Therefore, embryo gender identification is mainly used for embryos before implantation, requiring shorter cycles than those of natural delivery identification. The findings of this study indicate that biopsy does not significantly affect the quality or viability of embryos. The established ultra-trace gDNA amplification system, in conjunction with *SRY*-specific probes, greatly enhances the sensitivity of identification and will be significantly beneficial for research on early mammalian embryonic development characteristics and sex control technologies. Furthermore, future research should continue to optimize and consistently monitor biopsy techniques meticulously, enabling the safe application of this method for sex determination and other genetic analyses in early embryos.

## 5. Conclusions

This study established a rapid, sensitive, and accurate method for identifying the sex of sheep embryos, which can be further used to identify the sex and species of other mammalian biological samples. This method combines ultra-low-temperature freezing and embryo transfer techniques to obtain offspring of the desired gender.

## Figures and Tables

**Table 1:** Primer sequences for sex identification.

Primers	Primer Sequences	Size of Products (bp)	Melting Temperature (°C)
*GAPDH* *SRY* *SRY*-Probe	Forward: GGTCCACATGGCCTCCAAG Reverse: TCCATTTGTGAGTGTGTGGTCTT Forward: CTATACACCGAGACAAATACCCG Reverse: AATCGTCCCTGTATGTGAAGG Forward: AM-AAGAGGCCACAGAAATCCCTTGCT-MGB (Minor Groove Binder)	167 148	65 60

## Data Availability

The original contributions presented in the study are included in the article; further inquiries can be directed to the corresponding authors.
